# Iron-doped calcium phytate nanoparticles as a bio-responsive contrast agent in ^1^H/^31^P magnetic resonance imaging

**DOI:** 10.1038/s41598-022-06125-7

**Published:** 2022-02-08

**Authors:** Natalia Ziółkowska, Martin Vít, Richard Laga, Daniel Jirák

**Affiliations:** 1 Department of Diagnostic and Interventional Radiology, MR unit, Institute for Clinical and Experimental Medicine, Prague, Czech Republic; 2 Institute of Biophysics and Informatics, First Faculty of Medicine, Charles University, Prague, Czech Republic; 3 Faculty of Mechatronics Informatics and Interdisciplinary Studies, Technical University of Liberec, Liberec, Czech Republic; 4 Institute of Macromolecular Chemistry, Czech Academy of Sciences, Prague, Czech Republic

**Keywords:** Biophysics, Chemistry, Physics

## Abstract

We present the MR properties of a novel bio-responsive phosphorus probe doped with iron for dual proton and phosphorus magnetic resonance imaging (^1^H/^31^P-MRI), which provide simultaneously complementary information. The probes consist of non-toxic biodegradable calcium phytate (CaIP_6_) nanoparticles doped with different amounts of cleavable paramagnetic Fe^3+^ ions. Phosphorus atoms in the phytate structure delivered an efficient ^31^P-MR signal, with iron ions altering MR contrast for both ^1^H and ^31^P-MR. The coordinated paramagnetic Fe^3+^ ions broadened the ^31^P-MR signal spectral line due to the short *T*_*2*_ relaxation time, resulting in more hypointense signal. However, when Fe^3+^ was decomplexed from the probe, relaxation times were prolonged. As a result of iron release, intensity of ^1^H-MR, as well as the ^31^P-MR signal increase. These ^1^H and ^31^P-MR dual signals triggered by iron decomplexation may have been attributable to biochemical changes in the environment with strong iron chelators, such as bacterial siderophore (deferoxamine). Analysing MR signal alternations as a proof-of-principle on a phantom at a 4.7 T magnetic field, we found that iron presence influenced ^1^H and ^31^P signals and signal recovery via iron chelation using deferoxamine.

## Introduction

Magnetic resonance (MR), which comprises non-invasive spectroscopy (MRS) and imaging (MRI), is a widely used method in a number of medical settings and applications. Noted for its high resolution, excellent soft tissue contrast and non-ionising radiation, MR has distinct advantages over other in vivo imaging techniques. While the use of contrast agents and tracers makes the method more sensitive and specific, biocompatibility-related issues can arise^1^. A number of contrast agents have been proposed, notably non-toxic probes that are responsive to various biochemical stimuli, tissue-specific targeting and theranostics^2–4^. One of the most effective methods is non-hydrogen X-nuclei MR imaging, which provides complementary information to anatomical ^1^H-MR imaging and visualisation of physiological processes^5–7^, even on a cellular level^8^. Phosphorus is one of the more promising X-nuclei and the second most commonly used nucleus in biological MR studies. Interest in phosphorus MR stems from its ability to provide unique information on the composition of cell membranes, bioenergetics, oxidative phosphorylation, intracellular pH and magnesium levels^9–12^. Most common phosphorus-containing compounds measured by in vivo ^31^P-MR, like adenosine triphosphate (ATP) and phosphocreatine (PCr), can generate a chemical shift of 0 ± 30 ppm^13^. Phosphorus contains the stable natural monoisotope ^31^P, which possesses a non-zero magnetic moment (spin = ½) and a gyromagnetic ratio of 17.2 MHz T^−1^ (about 60% lower than that of ^1^H). Completely abundant in nature, phosphorous is detectable even at the low magnetic fields typically used in clinical practice. Positioning various paramagnetic (Mn^2+^, Fe^3+^ or Gd^3+^) or superparamagnetic (SPION) ions in the vicinity of the phosphorus nucleus can considerably affect relaxation times and, as a result, change the contrast properties of both ^1^H^14,15^ and ^31^P MR images. In cases of extremely short *T*_*2*_ relaxation times, the MR signal may disappear completely. In this article, we present a novel magnetic resonance contrast agent based on calcium phytate (CaIP_6_) nanoparticles doped with paramagnetic iron irons (Fe^3+^) (for the chemical properties, see^16^). Compared with other ions used in MR probes, such as Gd^3+^, the biogenic Fe^3+^ ion boasts highly regulated naturally occurring systems and ensures safe transport and storage throughout the organism^17^, thus reducing any long-term toxic effects.

The non-toxic nature of the probe is crucial for its application in medicine, including therapy^18^ and diagnosis. Some phosphorus-containing organic materials are considered highly biocompatible^19^ and thus suitable for medical use. One of the more promising of the phosphorus-based compounds for medical settings is phytic acid (IP_6_)^20^. Also known as myo-inositol-1,2,3,4,5,6-hexakisphosphate, IP_6_ is a fully phosphorylateed derivative of inositol that occurs naturally in plants, especially in seeds and grains. The profuse presence of phytate in biological systems means it is highly biocompatible^21^, as confirmed by the use of technetium-99 m (^99m^Tc-phytate) in in vivo diagnostics^22^. Comprising six negatively charged phosphate groups, IP_6_ is adept at capturing cations. It also inhibits iron‐mediated generation of the highly toxic hydroxyl radical^23^, chelating polyvalent metal cations and preventing its uptake in the digestive tract^24^. The resulting ablation of the system significantly reduces pathogen’s ability to colonise hosts^25^. This reduction in iron uptake is compensated for by siderophores^26,27^, which are compounds capable of capturing iron and distributing it to microbial cells^28^ and a number of pathogens during infection. Siderophores are relatively low molecular weight substances that act as chelating agents for iron ions produced by pathogenic, as well as non-pathogenic bacteria and fungi generated during iron deficiency. While iron-chelating siderophores produced by bacteria display high affinity for Fe^3+^ ions, nanosized siderophore-containing substances can also be used to accelerate the complexation of iron, as in a previous physiological stimulation model^16^, the iron-binding bacterial siderophore deferoxamine (DFOA), an FDA-approved small molecule drug used in iron overload treatment^29^ originating from the bacteria *Streptomyces pilosus*. Siderophores are also useful in diagnosing bacterial diseases^30–32^, including infections of the gastrointestinal tract and those associated with local-use implants. The iron-chelating ability of siderophores was also recently investigated in relation to malignant cancerous cells^33^ and in antibiotic-siderophore conjugates^34^. Low concentrations of calcium phytate nanoparticles doped with Fe^3+^ ions have been shown to be non-toxic, a precondition for in vivo application.

Due to the presence of paramagnetic Fe^3+^ ions, the nanoparticles act primarily as a *T*_*2*_ contrast agent in ^1^H-MR. Short relaxation times provide a hypointense contrast on *T*_*2*_-weighted ^1^H-MR images while simultaneously broadening the ^31^P peak leading the phosphorus MR signal to a noise level, making it impossible to detect at higher iron concentrations. On the other hand, the phosphorus MR signal recovers when Fe^3+^ concentration in the phytate complex is lower.

In this proof-of-principle phantom study, we investigated the effect of different iron concentrations on the proton and phosphorus MR signals of calcium phytate at a 4.7 T magnetic field. We also evaluated the MR sensitivity and MR properties of the probe, tested its biocompatibility and visualize recovery of the MR signal in a chelation model using the iron-binding bacterial siderophore DFOA.

## Results

### ^1^H/^31^P magnetic resonance coil homogeneity

**Figure 1:**
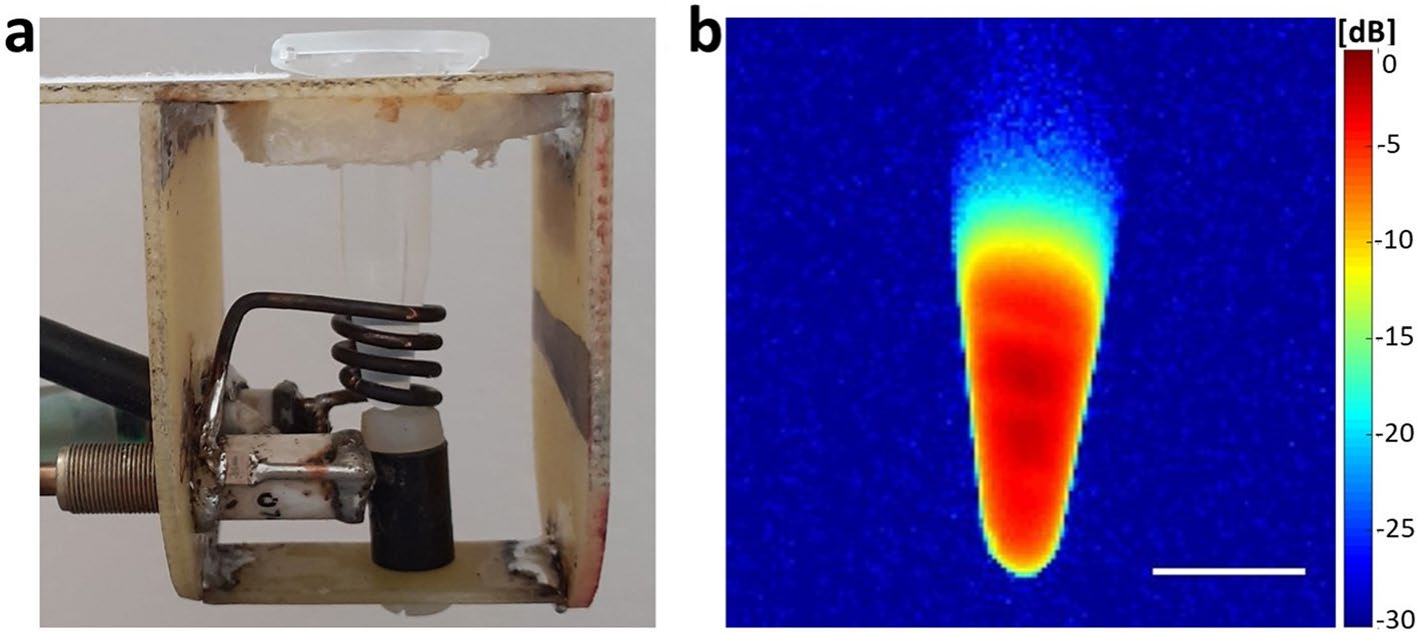
Homogeneity of ^1^H/^31^P radiofrequency solenoid coil measured using a water phantom and ^1^H-MRI on a 4.7 T scanner: (**a**) ^1^H/^31^P radiofrequency solenoid coil with 500-µL tube suitable for high SNR measurement; (**b**) axial plane, with scale bar representing 10 mm; signal intensity is represented by a colour scale (dB) reflecting signal attenuation—from red (highest signal) to blue (lowest signal).

A field of view providing sufficient image quality was determined for further implementation of the coil. The working area of the radiofrequency (RF) coil is presented with signal attenuation (∆5 dB) to indicate areas of high sensitivity measurement. Inhomogeneity in the image, which reflects the positioning of the RF conductors, is characteristic of solenoid coils. The coil enables high signal-to-noise (SNR; Eqs. 1 and 2) measurement, which is crucial for ^31^P MR at low magnetic fields. The working area and sensitivity of the RF coil are visualised in Fig. 1.

### ^1^H magnetic resonance of phantoms

**Figure 2:**
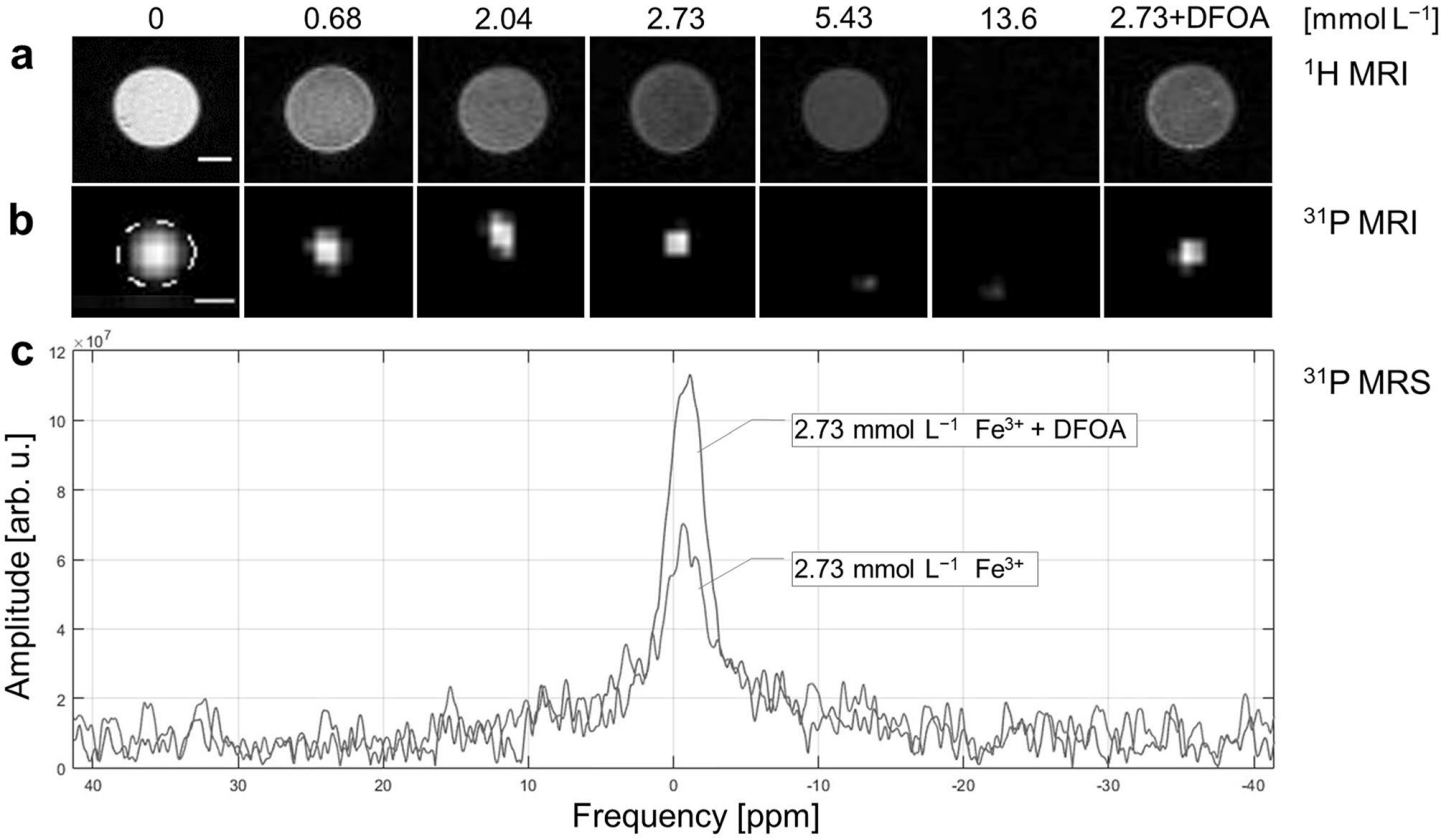
MR results for phantoms at different iron doping concentrations measured on a 4.7 T scanner: (**a**) ^1^H-MRI and (**b**) ^31^P-MRI for calcium phytate nanoparticles doped with 0–13.6 mmol L^−1^ Fe^3+^ and 2.73 mmol L^−1^ Fe^3+^ probe with DFOA, with scale bar representing 10 mm and dotted line showing quantified region of interest for phosphorus signal; (**c**) ^31^P-MRS comparison of 2.73 mmol L^−1^ Fe^3+^ probes with and without DFOA chelation.

**Figure 3:**
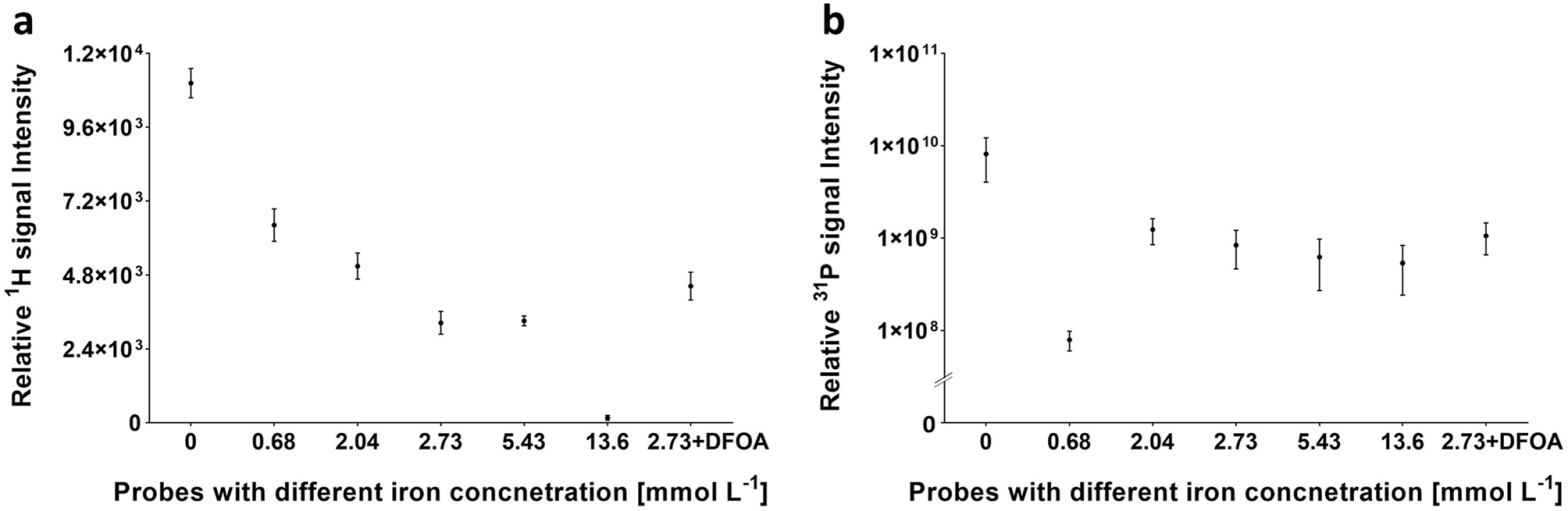
Relative (**a**) ^1^H-MRI and (**b**) ^31^P-MRI signal intensity (SI) dependence of calcium phytate probes doped with different iron concentrations (*c*^Fe^ = 0–13.6 mmol L^−1^) measured on a 4.7 T scanner. Error bars represent standard deviation of mean signal intensity.

^1^H-MRI of phantoms containing high amounts of iron appeared darker on *T*_*2*_-weighted images compared to images with lower iron content (Fig. 2a). The lowest SNR = 1.2 issued from the probe doped with the highest Fe^3+^ concentration and, as expected, the highest SNR = 86.2 issued from the probe containing no iron. Reflecting the effect of iron chelation, the probe with added deferoxamine (DFOA; 2.73 mmol L^−1^ Fe^3+^ + DFOA: SNR = 34.7) provided a 36%-higher ^1^H-MR signal compared to its pre-chelation form (2.73 mmol L^−1^ Fe^3+^: SNR = 25.4). The sensitivity curve reflected the signal intensity (SI) of MR dependence on iron concentration (Fig. 3a). The ^1^H image contrast was affected even at low iron concentrations (starting with 0.68 mmol L^−1^ Fe^3+^), confirming the effectiveness of the probe as a *T*_*2*_ contrast agent. Imaging results were supported by relaxometry findings; both *T*_*1*_ and *T*_*2*_ relaxation times decreased with increasing iron concentration, resulting in *T*_*2*_ = 22.2 ms for 2.73 mmol L^−1^ Fe^3+^ and *T*_*2*_ = 33.9 ms for the iron-free probe. Results are summarised in Fig. 2, Tables 1 and 2.

**Table 1:** Signal-to-noise ratios (SNR) from ^1^H, ^31^P-MRI and ^31^P-MRS measured on a 4.7 T scanner.

Fe [mmol L^−1^]	SNR from ^1^H MRI	SNR from ^31^P MRI	SNR from ^31^P MRS
0	86.2	41.8	45.9
0.68	50.1	4.8	10.9
2.73	25.4	3.0	13.0
5.43	25.9	1.8	NA
13.6	1.2	1.8	NA
2.73 + DFOA	34.7	4.3	72.2

*NA* not applicable.

**Table 2:** ^1^H (1.5 T) and ^31^P (4.7 T) *T*_*1*_/*T*_*2*_ relaxation times [ms] ± standard deviation.

Fe [mmol L^−1^]	^31^P *T*_*1*_ [ms]	^1^H *T*_*1*_ [ms]	^31^P *T*_*2*_ [ms]	^1^H *T*_*2*_ [ms]
0	1278.7 ± 12.1	122.3 ± 0.5	121.3 ± 3.0	33.9 ± 0.7
0.68	277.9 ± 40.4	111.7 ± 1.0	22.1 ± 0.8	26.4 ± 0.4
2.73	266.7 ± 22.7	101.3 ± 0.6	19.2 ± 1.5	22.2 ± 1.1
5.43	NA	88.0 ± 0.0	NM	19.9 ± 1.0
13.6	NA	69.3 ± 0.8	NM	18.7 ± 3.6

*NA* not applicable, *NM* not measured.

### ^31^P magnetic resonance of phantoms

The ^31^P-MRI signal with the highest SNR = 41.8 in the iron-free probe decreased as iron content in the probe increased (Fig. 2b) until the signal was no longer detectable at 5.43 mmol L^−1^ Fe^3+^. Additionally, coordinated iron ions affected ^31^P-MRS signal broadening in correlation with increasing Fe^3+^ concentration; no signal was obtained from the highest concentrations ≥ 5.43 mmol L^−1^ Fe^3+^ in accordance with ^31^P and ^1^H-MRI results. Iron chelation was visible in ^31^P-MRS of the 2.73 mmol L^−1^ Fe^3+^ probe with added DFOA, showing an increased signal (SNR = 72.2) after iron ion decomplexation by the siderophore compared to the signal without DFOA (SNR = 13.0; Fig. 2c). Signal restoration was also observed in ^31^P-MRI, with the DFOA-added probe recording an increase in signal intensity (SNR = 3.0 vs 4.3). As shown in the sensitivity curve for the relative ^31^P-MRI signal intensity (Fig. 3b), the addition of DFOA increased signal detectability by 26%.

The ^31^P *T*_*1*_ relaxation times experiment required a long acquisition time (up to 150 min) due to low phosphorus sensitivity, with the sedimentation of nanoparticles affecting measurement results. Nonetheless, ^31^P relaxometry confirmed the iron effect, where the relaxation time decreased with iron doping (*T*_*1*_ = 1278.7–277.9 ms; 0–0.68 mmol L^−1^ Fe^3+^) and *T*_*1*_ = 266.7/229.1 ms for the 2.73 mmol L^−1^ Fe^3+^ with or without DFOA. The ^31^P *T*_*2*_ relaxation time for the iron-doped probe was also shorter (2.73 mmol L^−1^ Fe^3+^; *T*_*2*_ = 19.1 ms) than for the probe without iron (*T*_*2*_ = 121.3 ms). Results are summarised in Fig. 2, Tables 1 and 2.

### Cell viability assay

**Figure 4:**
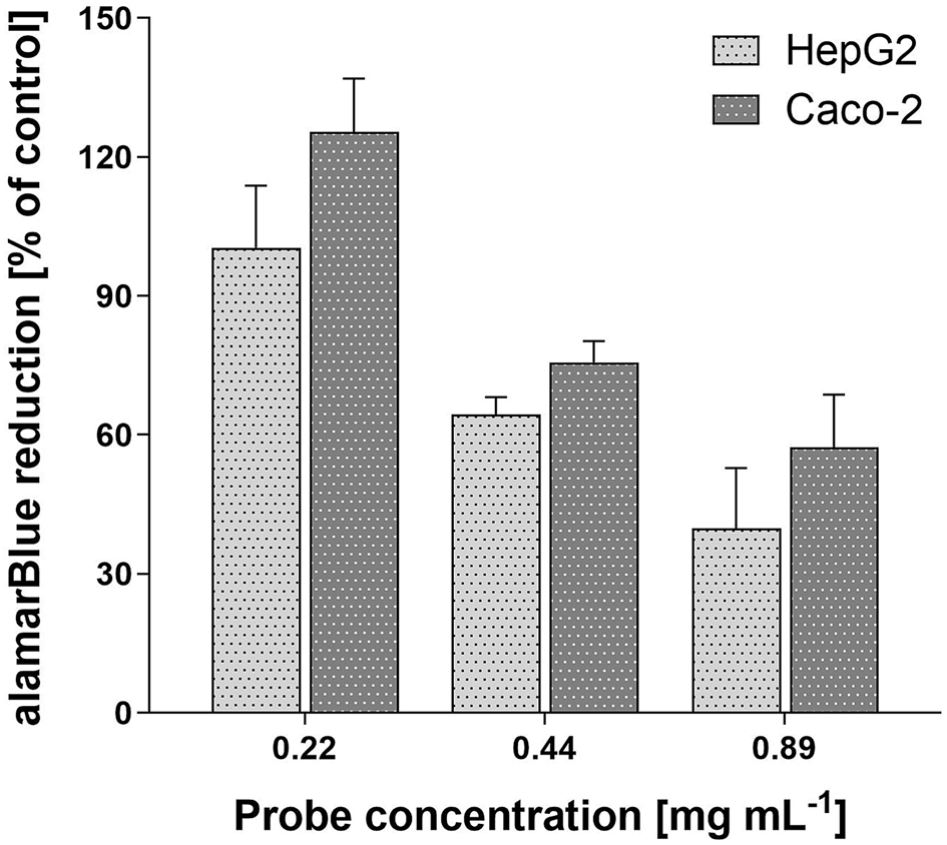
Results of alamarBlue cell viability assay, represented as a percentage of the resazurin reduction by HepG2 and Caco-2 cells comparing to the control cells after a 24-h incubation with the 2.04 mmol L^−1^ Fe^3+^ probe with the calcium phytate concentration of 0.22–0.89 mg mL^−1^.

Based on alamarBlue Assay, the 2.04 mmol L^−1^ Fe^3+^ probe did not influence cell viability, avoid exceeding 0.22 mg mL^−1^ ([2.04 mmol L^−1^ Fe^3+^]:[cultivation medium] ratio 1:3). The level of absorbance at 570 nm reflected the level of cell proliferation. Compared to control cells, Caco-2 incubated with a 0.22 mg mL^−1^ solution of the probe in growth medium resulted in a 125.5 ± 11.5% reduction of resazurin after 24 h. Under the same conditions, HepG2 cells attained a 100.4 ± 13.4% reduction. It should be noted that even low concentrations of the probe are suitable for MR analysis, decreasing any potential toxic influence of iron ions. Results are summarised in Fig. 4.

## Discussion

The probe characterization^16^ stated that TEM micrographs of the probes resembles roundish polydisperse primary particles with sizes of 50–100 nm, which formed loose aggregates of micron sizes, but the sizes measured with DLS correspond to none of the structures observed with TEM. Based on this finding it was suggested that in colloidal dispersions nanoparticles exist as aggregates of several primary particles. Moreover, the number average diameter of the nanoparticles was measured using DLS and was proved to be similar before and after addition of DFOA. The long-term stability of the nanoparticles was supported by repeatable ^31^P-MR spectroscopy with no spectral line separation or half-width change. The particle size is close to the nanoparticles in biodegradable system that has been clinically approved^35^. The biodegradation and accumulation of the Fe^3+^ in the in vivo environment was discussed in Fe^3+^-doped silica nanoshells, which were found to degrade via removal of the iron by serum proteins, such as transferrin, over a period of several weeks, while submerged in fetal bovine and human serums at physiological temperature^36^. Furthermore, in our probe, phytic acid chelates metal cations, such as Fe^3+^, and prevents their uptake in the digestive tract. Calcium phytate is non-toxic and naturally abundant substance^21^, which is indigestible by humans, so the nanoparticle can pass through gastrointestinal tract without degradation and be cleaved out from the organism. We are aware, that in vivo accumulation should be investigated more and experiments on probes’ pharmacokinetics and metabolism should be conducted in the future.

Acquisition of a reproducible and quantifiable MR signal requires a homogeneous magnetic field throughout the sample. The custom coil presented in this article, which is designed for use in MR experiments, meets these homogeneity requirements, generating limited signal attenuation (∆5 dB) and high sensitivity for such a small volume of interest at 4.7 T MR. Moreover, we assume the signal can be further amplified to improve the SNR using ^1^H RF part for nuclear Overhauser enhancement and ^31^P-^1^H decoupling or the quadrature ^31^P circular polarisation system. From our previous experience, we can say that the tested solenoid coil provided up to 40 times higher sensitivity than commercial surface coils at a diameter of 40 mm. We found that with increasing iron concentrations in the probe, both ^1^H and ^31^P relaxation times shortened. However, our ^31^P-MR results produced inconsistencies, with signals proving insufficient for quantification possibly due to lower calcium phytate nanoparticle colloidal stability. This can be accompanied by sedimentation during long measurements, which may result in low SNR and sensitivity at the 4.7 T magnetic field strength. Nonetheless, the adequate iron/phosphorus concentration achieved would make it suitable for use even at the lower magnetic fields used in clinical practice, like 3 T scanners. With regard to hydrogen relaxation times, short-time acquisitions at 1.5 T relaxometer served to decrease the influence of sedimentation. High concentrations of phytate may also have impact on quantification, with relaxation times reducing even in the absence of iron. This may in turn have affected the phosphorus *T*_*1*_ relaxation time at 4.7 T recorded for the 2.73 mmol L^−1^ Fe^3+^ probe after chelation, which was slightly shorter after the addition of DFOA (data not shown). However, SNR after chelation increased in respect of both ^1^H/^31^P imaging and ^31^P spectroscopy. The quality of ^31^P and ^1^H-MR images, as well as ^31^P spectra, are reflected by SNR calculation.

We found that iron had a sufficient influence on ^1^H-MR contrast, making the probe suitable for the use at low iron concentrations similar to those used for clinically approved contrast agents, e.g. Resovist with *c*^Fe^ = 0.45–0.7 mmol L^−1^^37^. As the iron molar fraction primary changed the *T*_*2*_ relaxation time of the probe (r_1_ = 7.2 L mmol^−1^ s^−1^, r_2_ = 17.5 L mmol^−1^ s^−1^; r_2_/r_1_ = 2.43), one can assume it acts as a *T*_*2*_ contrast agent. In comparison, other iron-based contrast agent, Ferumoxytol, has r_1_ and r_2_ relaxivities of 10 L mmol^−1^ s^−1^ and 62.3 L mmol^−1^ s^−1^, respectively^38^. This effect of iron on MR signal indicates that we can expect high contrast between the probes containing the iron and surrounding tissue in future in vivo applications. Signal detection decreased in correlation with increased iron concentration for both ^1^H and ^31^P-MR. The probes therefore possess double contrast capability, influencing both ^1^H and ^31^P-MR modalities in a similar way. Our data indicate that samples with high iron content would be less practical, as high concentrations of ferric ions can saturate siderophores’ binding capacity or prolong iron decomplexation, which will result in extended experiment time and lower sensitivity.

Therefore, we concluded that the sample with lower iron content, although sufficient for signal broadening, would be beneficial for signal restoration by siderophores. To test signal restoration under conditions mimicking bacterial infection, DFOA was added to the phytate probe containing 2.73 mmol L^−1^ Fe^3+^. Even though the DFOA/Fe^3+^ complex signal did not reach the value of the iron-free probe, signal restoration was visible in MR results. Using both modalities, the sample was adept at observing signal changes in dual ^1^H/^31^P contrasts. Additionally, complexed iron was distant enough from the phosphorus atoms, in agreement with previous results by Pechrova et al.^16^, so Fe^3+^ released from the nanoparticle and complexed with DFOA had a limited effect on the ^31^P-MR signal, despite slightly reducing relaxation times. The DFOA siderophore, a potent chelator of iron ions, proved sufficient at low iron concentrations. Phytate is also known to chelate iron^39^ by lowering its absorption in the gastrointestinal tract, even though gastric acid lowers pH in the proximal duodenum and enhances the solubility and uptake of ferric iron, and transferrin with ferritin bind circulating plasma iron^40^. This competition can be mitigated by changing phytate/Fe^3+^ concentrations depending on the injection site, given that siderophores are secreted under iron-stress conditions in gut microbiota. Furthermore, phosphorus imaging in the bacterial-overload can be used to track iron chelation rate in antibiotic-siderophore conjugates, which are more susceptible in different bacteria strains than those with the unlinked drugs^34^. The nanometer-sized range and amorphous nature of the particles accelerate iron complexation by siderophores. When targeting the probe, the background of the naturally occurring ^31^P-MR signal from the organism should also be considered.

Cytotoxicity assay results confirm that our novel probe exhibits no toxicity for low concentration. The results obtained for higher concentrations are possibly triggered by the presence of iron and obstacles with sedimentation. This should not be relevant in in vivo use in gastrointestinal tract with its pH and adaptation, which may be considered at low-risk when exposed to our probe. Especially, in comparison with cell culture testing and considering phytate native nature and low iron concentrations. Furthermore, small iron concentration use would eliminate its overload and support iron-mining bacteria. In addition, it has been demonstrated that the amount of iron can be adjusted, with probes containing low quantities of Fe^3+^ ions also providing sufficient contrast.

## Conclusion

We present one of the first phosphorus-based responsive contrast agents for ^1^H and ^31^P magnetic resonance. In our study, MR relaxometry, imaging and spectroscopy all confirmed the probe to be of sufficient sensitivity at a magnetic field similar to that used in clinical practice. In both spectroscopic and imaging experiments, the probe proved responsive to iron chelation with deferroxamine, which mimicked the presence of bacterial siderophores. It can therefore be assumed that ^31^P-MR signal changes could serve as a marker in reflecting the presence of pathogens. This phosphorus-based contrast agent is exhibiting no cytotoxicity at 0.22 mg mL^−1^, when compared with control cells, what indicates it is suitable for in vivo application.

## Materials and methods

MR experiments using calcium phytate phantoms were performed on the Bruker Biospec 47/20 4.7 T MR experimental scanner (Bruker Biospec, Ettlingen, Germany) and the 1.5 T Minispec 60 MHz relaxometer (Bruker Biospin, Ettlingen, Germany). MR properties of nanoparticles were determined using MRI, MRS and relaxometry. The chemical and NMR (Bruker Avance DPX 300 NMR) properties of the probe have been previously described (see^16^).

### Calcium phytate nanoparticles

Nanoparticles are based on calcium phytate (CaIP_6_; *c*^n^ = 0.89 mg mL^−1^) with a phosphorus concentration (*c*^P^) of 1 mmol L^−1^ in all probes. The distinguishing factor between the probes is doping with different paramagnetic Fe^3+^ ion concentrations (*c*^Fe^ = 0, 0.68, 2.04, 2.73, 5.43, 13.6 mmol L^−1^) coordinated to the phytate anion by partial replacement of Ca^2+^ ions, as previously described^16^. Calcium phytate nanoparticles were synthetised by mixing an aqueous solution of calcium nitrate (*c* = 1 mol L^−1^; adjusted by 1 mol L^−1^ NaOH to a final pH of 8) with an equal volume of an aqueous solution of sodium phytate (*c* = 0.16 mol L^−1^, pH 8). Doping of calcium phytate nanoparticles with iron ions was achieved by mixing an aqueous solution of calcium nitrate (*c* = 1 mol L^−1^) with an aqueous solution of FeCl_3_ (*c* = 1 mol L^−1^) at a different ratio prior to adding the IP_6_ solution; otherwise, the preparation procedure was the same as mentioned above. Hydrodynamic diameters d_N_ of neat CaIP_6_ particles was slightly above 100 nm with the highest value of 439 nm for 5.43 mmol L^−1^ Fe^3+^ dopant^16^. The particle size and ξ-potential were determined by dynamic light scattering (DLS). Simulation of Fe^3+^ release was performed by incubating CaIP_6_/Fe_3+_ nanoparticles with DFOA ([DFOA]:[CaIP_6_ with 2.73 mmol L^−1^ Fe^3+^] ratio 1:1; 2 h incubation), a bacterial siderophore with high affinity for Fe^3+^ ions. For testing, a 2.73 mmol L^−1^ Fe^3+^ probe was used. A sufficient iron concentration was generated for phosphorus MR signal broadening (see 3.3.) while avoiding over-chelation. The integrity of calcium phytate nanoparticles after the complexation of iron was proven (number average diameter of calcium phytate nanoparticles measured before addition of DFOA and 2 h after addition was 272 ± 41 nm and 288 ± 59 nm, respectively^16^).

Phantoms for MR imaging and spectroscopy were tested at all iron concentrations and prepared in 500-µL Eppendorf tubes (Eppendorf AG, Germany) and in NMR tubes for ^1^H relaxometry. Due to its tendency for sedimentation, calcium phytate was mixed repetitively using a vortex mixer before all measurements.

### ^1^H/^31^P magnetic resonance radiofrequency coil

The MR signal was acquired using a custom-made dual ^1^H/^31^P RF 4-turn one-channel solenoid coil (diameter = 8 mm, height = 8 mm). The volume of interest in the coil cavity was 400 µL, with the coil structure designed for 500-µL tubes. The coil was run at 200 and 81 MHz frequencies (^1^H and ^31^P, respectively), with retuning performed on a rotating capacitive trimmer (2–64 pF). A matching capacitive trimmer (4–16 pF) was incorporated to suppress waves reflected in the cable, which can cause signal attenuation. Coil homogeneity was measured on a water phantom (500-µL tube filled with H_2_O) by ^1^H-MRI, assuming homogeneity of the phosphorus signal was comparable. For homogeneity assessment, we used a gradient echo with a low flip-angle (FLASH) sequence (repetition time/echo time TR/TE = 111.7/3.7 ms, 10° flip angle, 256 × 256 matrix, field of view FOV = 40 × 40 mm, slice thickness = 2 mm, scan time ST = 28 s).

### Magnetic resonance relaxometry

Hydrogen *T*_*1*_ and *T*_*2*_ relaxation times were evaluated using phantoms with different iron concentrations (*V* = 240 µL; *c*^P^/*c*^Fe^ = 1/0–13.6 mmol L^−1^) measured at a stable 37 °C temperature. All data points were measured as the average of three repetitions. An inversion recovery sequence (TR = 0.01–10 000 ms, TE = 0.05 ms, recycle delay = 2 s, 1 scan, 8 points for fitting) was used to measure *T*_*1*_ relaxation times. *T*_*2*_ relaxation times were measured using the Carr-Purcell-Meiboom-Gill sequence (TR/TE = 10 000/0.05 ms, recycle delay = 2 s, 8 scans, 20 000 points for fitting). Low scan numbers and data points, which resulted in short acquisition times, were applied to eliminate the influence of sedimentation. ^1^H *T*_*1*_ and *T*_*2*_ relaxation times were fitted automatically using Minispec software (minispec NF, version 8.0, Bruker, Germany).

Phosphorus *T*_*1*_ relaxation times (*V* = 500 µL; *c*^P^/*c*^Fe^ = 1/0–13.6 mmol L^−1^) were measured based on 10 spectroscopic single-pulse sequences with varying repetition times (TR = 200–3000 ms, ST = 10–150 min). ^31^P *T*_*2*_ relaxation times (*V* = 500 µL; probe without iron and with 2.73 mmol L^−1^ Fe^3+^) were measured based on 10 spectroscopic Carr-Purcell-Meiboom-Gill (CPMG) sequences with varying echo times (TR = 5000 ms, TE = 2–1200 ms, ST ~ 90 min).

### Magnetic resonance imaging and spectroscopy

Hydrogen MR imaging (FLASH sequence; TR/TE = 100/6 ms) was first applied for phantom positioning. All phantoms (*V* = 500 µL; *c*^P^ = 1 mmol L^−1^) were measured at different Fe^3+^ concentrations (*c*^Fe^ = 0–13.6 mmol L^−1^) as part of one measurement using a custom-made holder and surface ^1^H/^31^P coil (area of interest 40 × 40 mm). To visualise the influence of iron on the ^1^H MR signal, *T*_*2*_-weighted ^1^H-MRI (Rapid Acquisition with Relaxation Enhancement RARE sequence; TR/TE = 2000/24 ms; scan time ST = 6 min 24 s, spatial resolution 0.25 × 0.25 × 1.5 mm) was used.

Phosphorus MR imaging of phantoms (*V* = 500 µL; *c*^P^/*c*^Fe^ = 1/0–13.6 mmol L^−1^) was carried out using MR spectroscopic imaging (MRSI chemical shift imaging CSI sequence; TR/TE = 500/15 ms, ST = 60 min, spatial resolution 2.5 × 2.5 × 5.8 mm). A non-localised single-pulse sequence was used to obtain ^31^P spectra of the probe chelated with DFOA (before/after chelation; TR = 500 ms, ST = 16 h 40 min).

### Magnetic resonance data quantification

MR imaging and spectroscopy data were quantified by calculating the SNR in order to determine the influence of iron on the MR signal. ^1^H and ^31^P-MRI signal intensities of phantoms were acquired by manual segmentation using ImageJ software (https://imagej.nih.gov/ij/, version 1.46r, National Institutes of Health, Bethesda, USA). ImageJ was also used for phosphorus MR images reconstruction. The SNR was calculated as a ratio of the signal intensity and standard deviation of the surrounding noise (Eq. 1):

1 $$I_{{\text{MRI}} \, {\text{SNR}}}=0.655\frac{S}{\sigma {\text{s}}}$$

where S is the signal intensity in the region of interest (ROI), σ is the standard deviation of background noise, with constant 0.655 reflecting the Rician distribution of background noise in the magnitude MR image^41^.

Phosphorus spectroscopic data were processed using Matlab software (https://mathworks.com, Matlab R2007b, The MathWorks, Inc., USA) script. Topspin software (ParaVision 4.0, Bruker, Germany) was used for ^31^P signal integral and noise calculation. The evaluation consisted of the following steps: the first was to mark the ^31^P peak to determine the integral of evaluation areas (∑I_signal_); the second was to select the noise region of the spectrum that is sufficiently shifted (∆7 ppm) from the peak to calculate the noise integral value (∑I_noise_). Signal integral value was used for ^31^P-MR spectra quantification of signal and noise. These values were used for SNR and *T*_*1*_*/T*_*2*_ relaxation times calculation. The SNR was calculated from the resulting values of the signal integral divided by the noise integral (Eq. 2):

2 $$I_{{\text{MRS}} \, {\text{SNR}}}=\frac{\sum {I}_{signal}}{\sum {I}_{noise}}$$

Finally, for ^31^P *T*_*1*_ and *T*_*2*_ relaxation times measurement, integrals from the evaluation area were fitted on exponential curves to the repetition time/echo time, respectively. ImageJ software was used for curve fitting followed by calculation of relaxation times. Signal intensity dependence on iron concentration and chelation was plotted to determine the effect of iron and DFOA on signal intensity changes for both ^1^H and ^31^P-MRI.

### Cytotoxicity assay

Probe cytotoxicity was tested on the human hepatoma cell line (HepG2; a standard in vitro testing model) and the human colorectal adenocarcinoma cell line (CaCo-2; replicating the gastrointestinal tract environment in which iron complexation can occur) to establish viability of the cells for use in in vivo experiments. The assay was tested using a 2.04 mmol L^−1^ Fe^3+^ probe—with a sufficient iron concentration for MR studies, while ensuring low iron impact on cell viability—at three CaIP_6,_ concentrations of 0.22–0.89 mg mL^−1^ representing 25–100% of the 2.04 mmol L^−1^ Fe^3+^ probe.

Cells were cultured in a standard humidified atmosphere containing 5% CO_2_ and 37 °C. CaCo-2 cells were grown in full high-glucose Dulbecco’s modified Eagle’s medium (DMEM, Thermo Fisher Scientific, USA) supplemented with 4.5 g L^−1^ d-glucose, 10% foetal bovine serum, 0.5% penicillin/streptomycin and 4 mmol L^−1^ l-glutamine. For HepG2 cells, a full Roswell Park Memorial Institute medium (RPMI, Thermo Fisher Scientific, USA 10% fetal bovine serum, 1% penicillin/streptomycin, 2 mmol L^−1^ l-glutamine) was prepared. alamarBlue Cell Viability Reagent (Thermo Fisher Scientific, USA) is a resazurin-based solution that functions as a cell health indicator and is used to reduce the power of living cells and quantitatively measure viability. Cells were seeded in 96-well plates at a density of 1 × 10^4^ cells/well, counted using the Vi-CELL XR Cell Viability Analyzer (Beckman Coulter, USA) and left to incubate for 24 h (HepG2) and 48 h (Caco-2). The phytate calcium probe was filtrated using a 0.2-μm filter, diluted in the fresh growth medium and added to the wells (100 μL/well). The effect of the contrast agent on cell viability was assessed based on 24-h incubation following 3-h (HepG2 cells) and 6-h (CaCo-2 cells) incubations with 10% alamarBlue. Resazurin, the active component of the reagent, was reduced to resorufin only in viable cells; absorbance in the test wells was detected at 570/600 nm using the Elisa Microplate Reader RT-6900 (Gen5 Software, BioTek, Germany). The alamarBlue reduction was then calculated as a percentage of the resazurin reduction in control cell. The assay was conducted in triplicate.

## Data Availability

All relevant data are included in the manuscript. Used datasets are available from the corresponding author on reasonable request.
